# Analysis of Indirect Biomarkers of Effect after Exposure to Low Doses of Bisphenol A in a Study of Successive Generations of Mice

**DOI:** 10.3390/ani12030300

**Published:** 2022-01-26

**Authors:** Francisca Bujalance-Reyes, Ana M. Molina-López, Nahúm Ayala-Soldado, Antonio Lora-Benitez, Rafael Mora-Medina, Rosario Moyano-Salvago

**Affiliations:** Departamento de Anatomía y Anatomía Patológica Comparadas y Toxicología, Facultad de Veterinaria, Campus de Rabanales, Universidad de Córdoba, 14014 Cordova, Spain; v02buref@uco.es (F.B.-R.); v22ayson@uco.es (N.A.-S.); v02momer@uco.es (R.M.-M.); r.moyano@uco.es (R.M.-S.)

**Keywords:** Bisphenol A, generations, rodents, blood biochemistry, biomarkers

## Abstract

**Simple Summary:**

Living beings are constantly and inadvertently exposed to a series of environmental and food pollutants, triggering effects on health that are transmitted over generations. Bisphenol A is a compound produced in large amounts world-wide and used in the manufacture of plastic containers and other utensils for daily use. It is an environmental and food pollutant with a demonstrated capacity to produce effects on the health of organisms exposed to it. The objective of our study was to identify possible indirect biomarkers of effect by means of the analysis of the blood biochemistry, and of certain reproductive parameters of animals exposed to Bisphenol A in doses considered to be safe over different generations. Our results did not show any modifications in the reproduction parameters evaluated, such as the duration of the estrous cycle, the size of the litters, or the percentage of the young alive at weaning time. However, they showed that there were alterations in biochemical parameters like glucose, total proteins, and albumin, which could therefore, be regarded as indirect indicators of an early effect of alterations in health caused by this compound.

**Abstract:**

Bisphenol A (BPA) is considered as being an emerging pollutant, to which both animal and human populations are continuously and inadvertently exposed. The identification of indirect biomarkers of effect could be a key factor in determining early adverse outcomes from exposure to low doses of BPA. Thus, this study on mice aims to evaluate and identify indirect biomarkers of effect through the analysis of their blood biochemistry, and of certain reproduction parameters after exposure to different BPA concentrations (0.5, 2, 4, 50, and 100 µg/kg BW/day) in drinking water over generations. Our results showed that there were no modifications in the reproductive parameters evaluated, like estrous cycle duration, litter size, or the percentage of the young alive at reaching the weaning stage, at the exposure levels evaluated. However, there were modifications in the biochemical parameters, e.g., alterations in the glucose levels, that increased significantly (*p* < 0.05) in the breeders at the higher exposure doses (50 and 100 µg/kg BW/day in F1; 50 µg/kg BW/day in F2 and 100 µg/kg BW/day in F3), that would suggest that the BPA could induce hyperglycemia and its complications in adult animals, probably due to some damage in the pancreas cells; albumin, that increased in the breeders exposed to the highest dose in F1 and F3, inferring possible hepatic alterations. Further, total proteins showed a diminution in their values in F1 and F2, except the group exposed to 100 µg/kg BW/day, whereas in F3 the values of this parameter increased with respect to the control group, this aspect likely being related to a possible hepatic and renal alteration. Based on these results, glucose, albumin, and total proteins could initially be considered as early indicators of indirect effect after prolonged exposure to low BPA doses over generations.

## 1. Introduction

Bisphenol A (2,2-bis[4-hydroxyphenyl]propane) (BPA) is one of the most studied endocrine-disrupting chemicals (EDCs), being one of the chemical compounds with the highest volume of world production, estimated as exceeding 10 million tons in 2022 [1]. The main uses of BPA are the manufacture of polycarbonate plastic and epoxy resins. Polycarbonate is used in the making of many everyday objects such as, spectacles medical equipment, mobile phones, consumer electronics, etc. Among the many uses for epoxy resins are industrial flooring, adhesives, industrial protective coatings, powder coatings, automotive primers, or food can coatings. BPA is also used in the manufacture of other food containers, being found as a food contaminant, due to, among other causes, migration from these containers [2,3]. Similarly identified as being a possible source of BPA exposure is thermal paper, in which this compound is used as a color developer, generating great concern as a potential source of contamination [4].

In 2017, the European Chemicals Agency (ECHA) classified BPA as a substance of very high concern due to its dangerous properties, so that the use of BPA is being limited in the EU to protect people’s health and the environment. In October 2019, ECHA recommended BPA to be included in the Registration, Evaluation, Authorization and Restriction of Chemicals (REACH) Authorization List (Annex XIV to REACH). BPA may damage fertility and has been identified as being a substance affecting the hormone systems of humans and animals. It is also listed in the EU as a substance that causes toxic effects on the human ability to reproduce (Repro. 1B), and possibly respiratory irritation (STOT SE 3), serious eye damage (eye dam. 1), and skin allergies (skin sens. 1) [5].

The ubiquity of this chemical compound, therefore, represents a widespread and continuous exposure of both animals and humans to it [6], with different routes of exposure such as diet, inhalation, or skin contact [7]. At an environmental level, it is an “emerging pollutant”, derived from the migration of packaging and waste dumped into the environment, which causes a serious problem of pollution in the terrestrial and aquatic spheres [8]. Despite having a short half-life in the environment of between 2.5–5 days [9], it has become a ubiquitous compound in the atmosphere, soil, surface waters, and sediments, as well as in free-living animals. Its presence has been widely studied in aquatic areas, being detected in surface waters in average concentrations of between 330 ng/L, groundwater (0–20 ng/L), wastewater, runoff water, and leachate, in which contamination by BPA would oscillate at levels of ng–µg/L [10,11].

Furthermore, BPA can coexist with other compounds in mixtures that can exert synergistic or additive effects on free-living species. The dose-effect and mode of action responses in BPA can vary between taxa and different stages of life, with some BPA metabolites being more estrogenic than the compound itself, and their characteristics can alter environmental degradation rates. Therefore, the environmental BPA effects are likely to be underestimated, since wildlife species could be exposed to higher concentrations of BPA in specific matrices such as leachate, river, and marine sediments, etc. [12]. Several studies show measurable effects of BPA on wildlife exposed to environmentally relevant concentrations (0.08 and 12.5 mg/L), which would imply that these populations are affected by environmental BPA [13]. In wild species, growing evidence suggests that an EDC, such as BPA, may interfere with sexual development in a wide variety of species [14]. Another potential risk of xenoestrogens (such as BPA) at the individual level in these species is the alteration of their reproductive function [15,16]. Understanding behavioral alterations among these free-living species could guide epidemiological studies in humans, in which such changes could serve as exposure gauges [17]. In summary, the concept of “One Health” could explain how the detrimental effects of BPA on different taxa in wildlife may provide key information on it in humans.

The European Food Safety Authority (EFSA) in 2015 published a global re-evaluation of the exposure and toxicity of BPA, reducing the tolerable daily intake (TDI) of BPA from 50 to 4 µg/kg BW/day. The TDI was temporarily established until BPA toxicity was reassessed following a biannual US National Toxicology Program (CLARITY BPA program), study aimed at conducting a primary toxicology study (regulatory toxicology) in conjunction with multiple behavioral, molecular, and cellular studies. The results of the main study would show that BPA does not cause any adverse effects according to the endpoints, or during the life of exposed animals of both sexes at below 25,000 µg/Kg BW/day [18]. Regulatory agencies nowadays state that BPA exposure is safe at the current exposure levels. However, many studies continue to show adverse effects in experimental animals exposed to low doses relevant to human exposure to BPA [19,20,21], which makes it necessary to continue with this type of research to carry out a new risk analysis.

To study BPA toxicity, multiple biomarkers of exposure and biomarkers of effect have been evaluated in different biomodels. Biomarkers of effect are measurable biological changes that help to establish dose-response ratios and mechanistic relationships. By providing a link between exposure, internal dose, and early health impairment, they could be extremely useful in improving Human Biomonitoring (HBM) and risk assessment of chemicals with a very short half-life such as BPA. The main biomarkers of effect for BPA are classified into those of molecular effect, such as malonaldehyde (MDA) indices in urine, indicators of oxidative stress, or expression of the KISS gene in placenta, determinants of reproductive disorders [22]. Regarding biochemical biomarkers of effect and parameters, such as testosterone levels (TT), estradiol, follicle stimulating hormone (FSH), and luteinizing hormone (LH) in serum provide information on enzymatic activity. Cortisol levels in serum and saliva have determined that BPA alters the hypothalamic-pituitary-adrenal axis. Serum glucose and insulin levels are a validated method of β-cell function and insulin resistance. Other biochemical parameters such as alanine aminotransferase (ALT), aspartate aminotransferase (AST), alkaline phosphatase (ALP-DEA), and lactate dehydrogenase (LD) are classic markers of liver damage [23,24].

On the other hand, the adverse effects of BPA on reproduction and development can result from early exposure to very low doses. The fetus and newborn represent populations especially vulnerable to exposure to EDCs since early development requires the precise timing of hormonal action to promote adequate tissue and organ growth. EDCs, specifically BPA, could interfere with the endogenous functions of these hormones. In addition, the enzymes involved in xenobiotic biotransformation and the elimination processes of these compounds are not fully developed in the fetus or neonate, thus BPA could persist and accumulate, reaching sufficient levels to cause adverse effects on the target organs in these populations [25,26]. Exposure to BPA early in life could have a transgenerational effect that predisposes later generations to the risk of developing a disease related to this endocrine disruptor.

The evident political and social preoccupation for the regulation and control of EDCs, and more specifically of BPA, includes new research objectives e.g., the development of new toxicological tests or the identification of new biomarkers of effect that clearly establish the risk estimation of this compound. Continued and inadvertent exposure to BPA makes it necessary to re-evaluate the possible effects of this compound at low doses and prolonged exposure. This preliminary study was designed to evaluate BPA toxicity at different exposure levels and in different generations by studying the alterations in biochemical and certain reproductive parameters that could be identified as early indirect indicators of effect over generations. To analyze these data, laboratory mice were used as an experimental biomodel that would provide general information on the effects of continuous exposure to different concentrations of BPA (0.5, 2, 4, 50, and 100 µg/kg BW/day) over several generations.

## 2. Materials and Methods

### 2.1. Animals, Breeding, and Housing Conditions

As an initial population, 24 mice C57BL/6JRj eight weeks of age with SPF (Specific Pathogen Free) health status supplied by Janvier Labs (Le Genest-Saint Isle, France) were used. Prior to the experimental phase, the animals were maintained for 10 days in an acclimatization period under identical environmental and housing conditions to those that were subsequently used in the experiment. The animals were kept under constant conditions of photoperiod (12-h light/dark cycle) and temperature (22–23 °C). The air changes in the room were constant (15–20 renewals/hour) and the relative humidity was maintained at 40–70%.

### 2.2. Study Design

**Figure 1:**
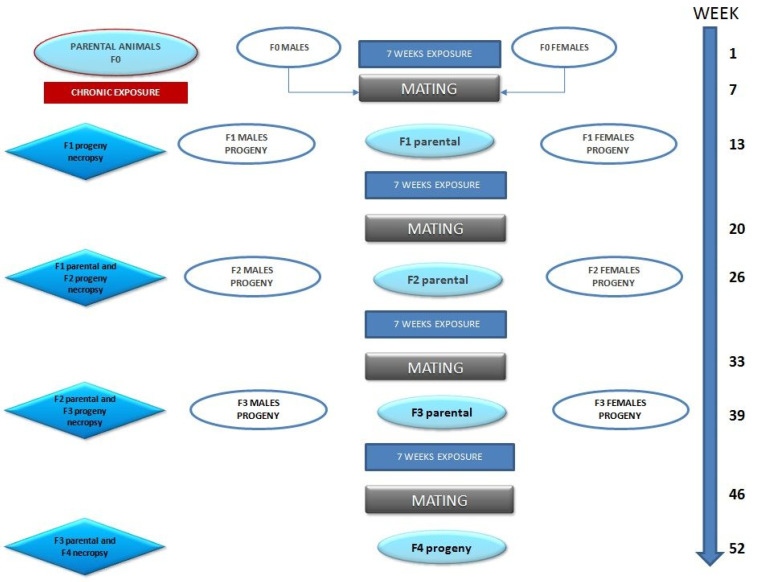
Study design.

The experimental design is shown in Figure 1.

After the acclimatization period, the animals were weighed and, according to their mean weight, randomly assigned to each of the study groups, according to the mean body weights per sex so all the groups were homogeneous at the beginning of the exposure period (Table 1). To establish generation 0 (F0), six groups were made (one control and five BPA exposure groups). The animals in the treated groups were exposed to a concentration of (0.5, 2, 4, 50, and 100 µg/kg BW/day) of BPA (Sigma Aldrich^®^, St. Luis, MO, USA) in drinking water. After an initial exposure period of seven weeks, matings were established between males and females of the same exposure group, confirming mating by the presence of a vaginal plug in the females in the morning. Once the pregnancy was confirmed, the animals were separated, and the females remained alone until the moment of delivery. After parturition, litter data were recorded, and at the end of the lactation period animals were randomly selected from the litters of each group as next-generation breeders. The F1 selected as the breeder began its period of exposure after weaning and for seven weeks after which the matings were established following the same process as in the F0. The experimental procedures were approved by the animal care committee of the University of Cordoba (Spain) (Authorization Code 26 June 2018/104) and conducted by the Experimental Animal Service, in accordance with European Regulations for the Protection of Experimental Animals (Directive 2010/63/EU).

### 2.3. Parent Animals; Experimental Evaluations

#### 2.3.1. Clinical Data of the Parent Animals (F0, F1, F2, and F3)

Routine observations were made daily through the cage throughout the study to detect clinical signs, morbidity and mortality, general appearance, and behavior. Likewise, a more detailed clinical evaluation was carried out outside the cage of all the animals once a week, making it coincide with the control of consumption and weight of the mice. In this evaluation, possible physical and behavioral anomalies of the study animals, such as changes in the coat, eyes, mucous membranes, secretions, postural alterations, or abnormal movements, as well as alterations in gait or abnormal behavior, were analyzed in greater detail. Food and water intake and the body weights of all the parent animals were measured weekly.

#### 2.3.2. Evaluation and Duration of the Estrous Cycle

The week before the scheduled mating, vaginal cytology was performed daily on the females in each group following a standard protocol. The sampling was carried out every 24 h for four consecutive days [27] at 8:00 a.m. with a sterile Henle loop, previously soaked in sterile saline. The vaginal epithelium samples were placed on slides and subsequently processed using Diff-Quick staining.

#### 2.3.3. Parental Reproductive Parameters

The reproductive parameters recorded were: the duration of the estrous cycle in days, the gestation duration calculated from day 0 (visualization of the vaginal plug), number of live-born pups per litter, percentage of males and females born, stillbirths, percentage of live pups at weaning, days until their eyes and ears were open, and breeding weights on days 0, 3, 7, 14, and 21 post-birth (PND0, PND3, PND7, PND14, and PND21), PND0 being considered the day of birth.

#### 2.3.4. Biochemical Analysis

The animals were anesthetized with isoflurane for blood extraction by intracardiac puncture to perform a biochemical analysis before their sacrifice by cervical dislocation. The biochemical determinations in the serum were carried out using the Atom A-15 automatic analyzer (Biosystems S.A., Barcelona, Spain), with kits from the same commercial company, measuring the following parameters: glucose (GLUC), urea (UREA), creatinine (CREAT), total cholesterol (TOT CHOL), triglycerides (TG), alkaline phosphatase (ALP-DEA), albumin (ALB), total protein (TOT PROT).

### 2.4. Progeny (F1, F2, F3, and F4); Experimental Evaluations

All live pups were counted and examined on the day of birth (designated PND 0) to determine the number of viable members of each litter. Thereafter, the litters were evaluated for survival on PND3, 7, and 14 and at weaning on PND21. All live F1, F2, F3, and F4 pups were individually weighed and examined for physical abnormalities at PND0, 3, 7, 14, and 21, the moment at which they were sexed.

#### Biochemical Analysis

At PND21, progeny was anesthetized again with isoflurane for blood extraction by intracardiac puncture, before their sacrifice by cervical dislocation, to perform a biochemical analysis of the following parameters: glucose (GLUC), urea (UREA), creatinine (CREAT), total cholesterol (TOT CHOL), triglycerides (TG), alkaline phosphatase (ALP-DEA), albumin (ALB), total protein (TOT PROT). The biochemical determinations in the serum were carried out using the Atom A-15 automatic analyzer (Biosystems S.A., Barcelona, Spain), with kits from the same commercial company.

### 2.5. Statistical Analysis

The results obtained were analyzed by IBM SPSS (version 25) employing different descriptive and inferential analysis techniques. The statistical techniques for proving our research hypotheses were selected by taking into account the nature of the variables, and the assumptions of normality and homoscedasticity. For the latter, the Kolmogorov and Levene tests, respectively, were applied.

Next, in order to see which factors influenced our principal research variables, linear models were applied, not only to prove the bivariate independence of the factors, but also to verify the multivariate independence between them and, therefore, in what way that factor interaction could influence our principal research variables.

## 3. Results and Discussion

BPA is a compound found in multiple consumer products and appears frequently in the environment, causing continued and inadvertent exposure of human and animal populations to it. Numerous studies have assessed the effects after exposure to BPA, using different biomodels. BPA has been seen to have a negative effect on different organic systems and functions such as reproduction [28], thyroid hormone [29], endocrine pancreas [30], immune system [31], adipose tissue, and pituitary function [32]. Certain EDCs could affect the function of the pituitary gland, affecting the synthesis and secretion of these hormones. Even at low doses, endocrine disruptors can exert toxicological effects, stimulating or inhibiting enzymes that play a fundamental role in hormone synthesis [33]. Some of these agents can inhibit specific enzymatic steps in the biosynthetic pathway of steroidogenesis. EDCs have been considered to be metabolism disruptors, with the liver and adipose tissue being their main target organs, in which they can cause, for example, adipogenesis. BPA is not capable of triggering fibroblasts to differentiate into adipocytes but does accelerate terminal adipocyte differentiation [34]. Findings suggest that in vivo prolonged exposure to BPA may increase adipose tissue mass and promote the development of obesity. Furthermore, BPA may cause changes in cholesterol and bile acid metabolism, as well as lipid dysregulation. There are reports that the perinatal exposure of mice and rats to BPA provokes an increase in the adipose tissue mass and hyperlipidemia [35]. An important characteristic of EDCs is the so-called minimum dose effect, or the non-monotonic dose response curve theory. This curve, that explains the behavior of many endocrine disruptors, is characterized by a slope that changes within the range of doses tried. Some curves are U-shaped, others are inverted Us, the trajectory of the curve can change in multiple points along the range of doses examined. In this sense, authors such as Takai et al. [36] found that a minimum concentration of BPA increased the development speed of embryonic cells, whereas doses 100,000 times higher decreased it.

With the aim of early-stage identification of possible indirect biomarkers of effect, the mouse was used as an experimental biomodel to study the effects of BPA on it after continuous exposure to low doses over successive generations. A wide variety of doses were considered of (0.5, 2, 4, 50, and 100 µg/kg BW/day) and selected in accordance with the current TDI (4 µg/kg BW/day) established by the EFSA. Other doses previously used in other studies such as 100 µg/kg BW/day [37], and lower doses of 0.5 and 2 µg/kg BW/day were also used [38]. Some authors have reported that lower concentrations would be enough to induce adverse effects in progeny as well as reproduction alterations in multigenerational studies. Exposure to the highest dose (100 µg/kg BW/day) during critical phases such as pregnancy had shown itself to have long-term harmful implications in the metabolism of some biochemical parameters such as glucose. That is why our study considered a wide variety of BPA doses, including the theoretical “safe levels” indicated by the regulatory agencies like EFSA.

### 3.1. Parent Animals

#### 3.1.1. Clinical Observations

The animals did not present any visible clinical alterations in any of the exposure groups of the different generations throughout the study period, which coincides with what was observed by other authors at these low dose levels of exposure [39].

#### 3.1.2. Increase in Weight, Feed, and Water Intake

The average daily weight gains and mean daily intakes both of food and drinking water are recorded in Supplementary Table S1. The data obtained, together with the evolution of weights throughout the exposure period, reflected values agreeing with those biologically usual for the age and physiological status of the animals (4–6 mL water and 4–5 g of feed daily). Higher weight gains and food consumption were noted in the females during the gestation and lactation periods that corresponded to weeks 8, 9, and 10 of exposure.

#### 3.1.3. Parental Reproductive Parameters

##### Estrous Cycle

**Figure 2:**
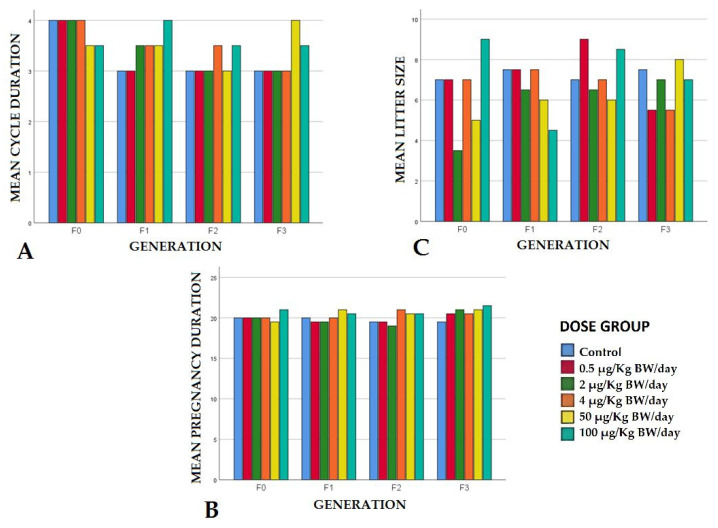
Mean reproductive parameter values per dose group and generation. (**A**) Cycle duration expressed in days; (**B**) Pregnancy duration expressed in days; (**C**) Litter size.

The reproductive females had normal cycles and the vaginal cytology did not reveal morphological abnormalities in any of the samples analyzed from any exposure group or any generation. The estrous cycle duration was biologically equivalent in all the groups exposed to BPA in relation to the control groups in all the generations, ranging from three to four days (Figure 2A). These results coincide with those obtained by other authors who, in reproductive toxicity studies in rodents exposed to concentrations of 50 µg/kg BW/day, did not find any abnormalities in the estrous cycle of reproductive females [40].

##### Pregnancy Duration

In our study, we evaluated whether exposure prior to conception at the doses proposed caused abnormalities in gestation duration; the latter’s mean values varied between a minimum of 19 days and a maximum of 21.5 (Figure 2B). Although the data display an increase in this gestation period in all the generations of the group, with the highest exposure level in the control group, and at low exposure levels of 0.5, 2, and 4 µg/kg BW/day, these values are within the biological ranges of gestation duration for this mouse strain (18–21 days). They are, therefore, regarded as being normal at these exposure doses. Other authors obtained significant increases for this reproductive parameter, but at much higher exposure doses than those evaluated here (3500 ppm) [39]. This fact may be relevant since, contrary to the results of these authors, exposure to phenols like BPA or BPS during pregnancy has been associated with preterm labor, although the potential effect of this chronic exposure on parents before conception is unknown. Epigenetic modifications produced by BPA in male and female gametes probably contribute to the etiology of premature labor. It is suggested that the period prior to conception is a critical phase, during which potentially adverse effects on pregnancy would increase [41].

##### Litter Size and Stillbirths

The mean values of the litter size ranged between maxima of nine pups (in the group exposed to 0.5 µg/kg BW/day in F2) and minima of 4.5 pups (in the group of 100 µg/kg BW/day in F1) (Figure 2C). Considering that the average size of the litters in this experimental model was of 6.53 pups per litter, these data are considered as being within biological normality. These results coincided with those obtained by other authors who exposed rodents to concentrations of between 2 and 20 µg/kg BW/day of BPA during gestation and did not observe any differences in the sizes of the litters obtained with respect to the controls in different generations [42,43].

##### Percentage of Pups Alive at Weaning

Survival rates at day 21 post-birth are reported in Table 2. Likewise, the number of offspring that were not alive at weaning corresponds to what is physiologically predictable for this mouse strain (80% survival at weaning). In addition, these weaning survival rate results coincide with those obtained by Rochelle et al. [39] who, in a range of doses of exposure to BPA including those used in this study, did not find any significant differences in weaning survival rates in a two-generation study in mice either.

##### Sex Ratio

Our results, presented in Table 3, show an alteration in the ratio male:female, with a higher percentage of males in the litters coming from the parental F1 exposed to 4 µg/kg BW/day. The litters from F1 and F2 exposed to 50 µg/kg BW/day, however, presented ratios in which female offspring prevailed over the control group. The percentage of males born in F3 exposed to the highest BPA concentrations (of 50 and 100 µg/kg BW/day), was higher than that of the females in the litters from F3, coinciding with authors such as Dobrzyńska et al. [44], who observed that the proportion of sexes was altered, accompanied by a decrease in sperm quality in generation F1 of mice, whose fathers (not mothers) were exposed for eight weeks, prior to crossing, to 5 and 10 mg/kg BW/day of BPA. They noted a prevalence of males over females, with respect to the control that was more marked at the highest exposure dose. However, in a study on two mice generations exposed to ranges of between 0.018 and 3500 ppm of BPA, a dose that resembled those used in our study, no effects related to that exposure or to the proportion of sexes at birth were found [39].

The alteration in this reproduction parameter is explained by the fact that, during spermatogenesis, equal amounts of X and Y spermatozoids are produced, but the proportion of sexual chromosomes in the sperm ejaculated could be altered due to the action of chemical substances with an endocrine-disrupting activity like BPA, which would be reflected in the proportion of the sexes at birth.

#### 3.1.4. Biochemical Parameters

Many studies have related exposure to BPA to a series of metabolic alterations that translate into an increase in body weight, obesity, insulin resistance, diabetes, and cardiovascular diseases, as well as liver and kidney alterations both in human and animal studies [45,46,47,48]. However, these effects vary significantly, depending on the age at exposure [49] and the dose. At another level, the effects and mechanisms of prenatal exposure, as well as the action mechanisms at low exposure doses in humans are poorly understood. In this study, biochemical parameters in serum that could be indirect indicators of the effect of BPA at low doses were analyzed in several mice generations to verify whether the metabolic disturbances resulting from that exposure are maintained over these generations.

The TG, GLUC, TOT CHOL, TOT PROT, CREAT, ALB, UREA, and ALP-DEA levels are presented in Table 4 for the breeding individuals. The statistical analysis of the data obtained from the breeder samples determined that there were significant differences (*p* < 0.05; *p* = 0.01) between the different groups and generations in the glucose and total protein levels in the blood, as well as in the albumin levels (*p* = 0.03). Blood triglyceride levels revealed differences (*p* = 0.05) between exposure groups and generations.

**Figure 3:**
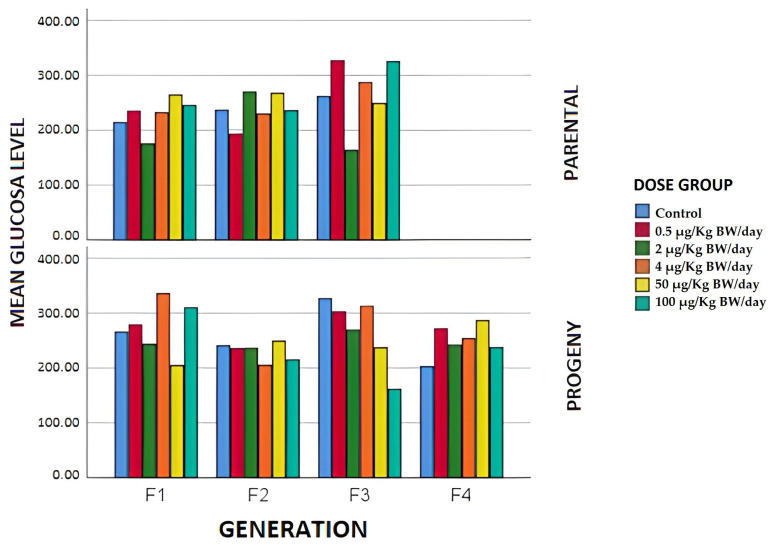
Mean glucose values expressed in mg/dL in the different generations of breeders and offspring in terms of the exposure group.

Regarding serum glucose levels in breeders in F1, all the exposure groups, except the 2 µg/kg BW/day group, presented higher glucose levels than the control group. The differences were more pronounced in the case of the breeders between the 50 and 100 µg/kg BW/day groups and the control, with the former giving higher mean values, whereas in the 2 µg/kg BW/day group the glucose levels were significantly lower. In the second generation of the study, however, blood glucose levels decreased compared to the control group in all exposed groups except the 2 and 50 µg/kg BW/day groups, in which this level increased with significant differences (*p* < 0.05). In the reproductive F3, the most notable differences occurred between the control and dose groups of 0.5 and 100 µg/kg BW/day, that presented significantly higher mean values. Thus, this hyperglycemic effect would be transmitted to generations after F0 and could increase with respect to the control, the non-monotonic behavior in the dose-response curve being evident, as can be seen in Figure 3, where the curve’s trajectory changes within the range of the doses tried, exhibiting multiple inflection points for all generations of breeders that is typical of EDCs and, especially of BPA. Based on these results it could be said that exposure to BPA would cause a hyperglycemic effect, possibly due to an abnormal metabolism of the glucose that is transmitted to later generations of breeders and that could be attributed to a likely induction of oxidative stress [50].

This non-monotonic behavior did not coincide with the results obtained by other authors, who exposed adult male mice to concentrations of 0.5 and 2 mg/kg BW/day of BPA for 4 weeks, verifying after this period that blood glucose levels increased in a dose-dependent manner [51]. Furthermore, other authors exposed 8-week-old rats for 8 weeks to doses of 5, 50, and 500 µg/kg BW/day and did not obtain any differences in plasma glucose levels between the control and the exposure BPA groups. They did not find any alteration in the function of pancreas β cells either [52]. This would show that at lower exposure concentrations, the body would still be able to compensate for the effect of BPA on the pancreas so that although there was an alteration in its function, no modifications in biochemical parameters were seen.

Numerous animal studies have shown that BPA exposure would lead to abnormal glucose metabolism [53], indicating that short-term treatment with BPA would produce metabolic abnormalities causing hyperinsulinemia and insulin resistance in mice. In humans, it has been widely demonstrated through epidemiological studies that there is a correlation between exposure to BPA and the development of chronic diseases including type 2 diabetes [54].

The mechanism through which BPA interferes with glucose metabolism is not completely clear, but estrogenic effects could be involved, varying according to the duration of exposure, the dose, the route, and the period of exposure. BPA is structurally similar to 17β-estradiol, and it binds to estrogen-related receptors (ER), such as ERα, ERβ, and ERγ, the G-protein-coupled estrogen receptor GPR30, and the peroxisome proliferator-activated receptor gamma (PPAR-γ). Although the mechanism of action is not fully understood, the binding of BPA to these receptors has been seen to induce insulin resistance, adipogenesis, pancreas β-cell dysfunction, inflammation, and oxidative stress [55]. Authors such as Marmugi et al. [56] demonstrated that exposure to BPA for eight months in adult mice produced a state of hyperglycemia, glucose intolerance, hypercholesterolemia, and increased cholesterol synthesis in the liver, with the development of dyslipidemia or impaired lipid metabolism.

**Figure 4:**
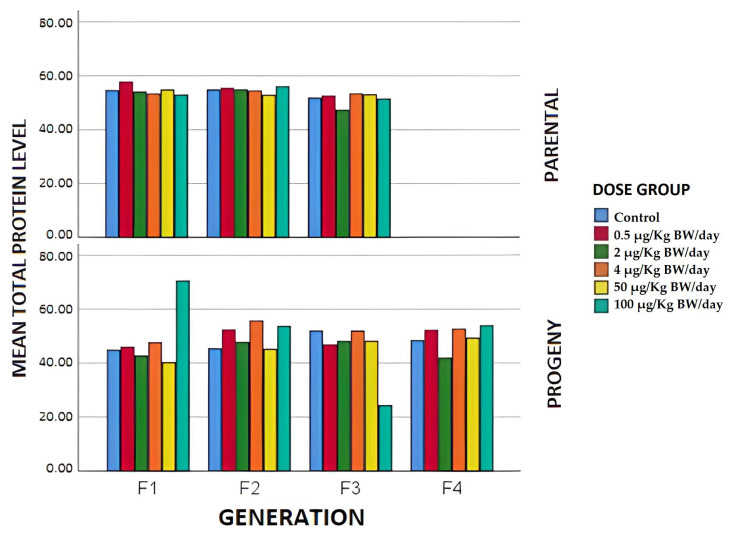
Mean total protein values expressed in g/L in the different generations of breeders and offspring in terms of the exposure group.

The total protein (TOT PROT) levels had lower mean values of this parameter than those of the control in all the exposure groups, except at the dose of 0.5 µg/kg BW/day in the F1. This is coincident with the results obtained by the Clarity BPA consortium, in which in its stop-dose study with 25 µg/kg BW/day there was a slight decrease in the level of total proteins in exposed males. As reported in Figure 4 in the case of F2, the same results were obtained, except for the group exposed to 100 µg/kg BW/day, in which the mean value was significantly higher than that of the control group. In F3, the mean values of total proteins were significantly lower in the 2 µg/kg BW/day group compared to those of the control group. In the remaining groups, this parameter tended to increase.

The serum protein level is a balance between the rate of protein synthesis and degradation. These total serum protein levels could be explained by considering that the bioavailable concentrations of estrogens are calculated by resolving the balance with serum proteins, and from among all of these, more importantly, albumin and steroid hormone binding globulins. However, scientific evidence has confirmed that exposure to BPA produces proteinuria, mainly related to the increase in urinary excretion of albumin, associated with hypertrophy in podocytes, and an increase in the glomerular filtration rate. Furthermore, the administration of BPA would alter liver integrity and functions [57]. The liver is regarded as being the main organ involved in the biosynthesis of plasma proteins; therefore, the reduction in the serum protein level would be indicative of liver damage, possibly induced by BPA, when the animals are continuously exposed to this compound.

This would have important implications from the point of view of xenobiotics´ toxicokinetics, since they tend to bind to plasma proteins to be distributed throughout the body. If there is a decrease in the level of plasma proteins, it would indicate that a larger number of unbound xenobiotics would remain, which is called the “free fraction or active fraction”, e.g., the one that could bind to specific receptors to trigger its toxic action and, therefore, generate harmful effects on the health of exposed animals.

**Figure 5:**
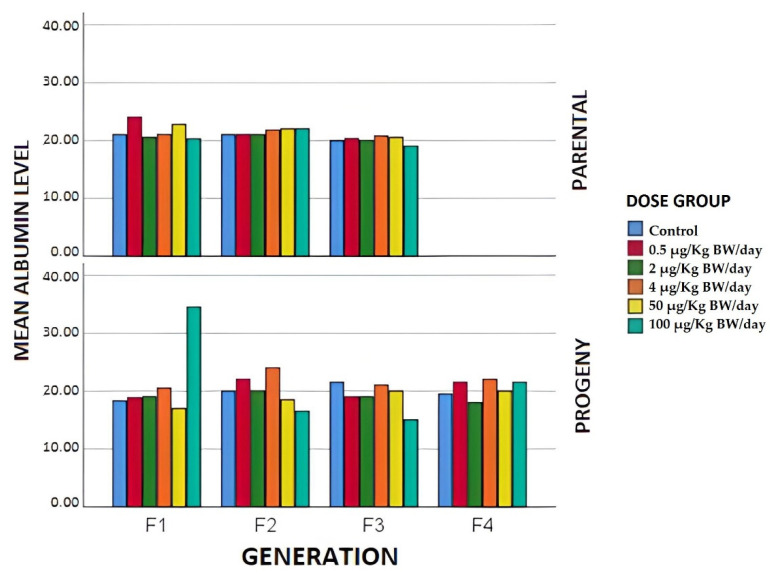
Mean albumin values expressed in g/L in the different generations of breeders and offspring in terms of the exposure group.

Regarding serum albumin, the results showed that in the case of first-generation breeders, Figure 5, there was an increase in the groups of 0.5 and 50 µg/kg BW/day, with respect to the control, while in the group exposed to the highest dose there was a significant decrease compared to the control group. The same result was obtained in individuals exposed to this higher dose in F3, which could be explained by possible liver damage.

These results agree with those obtained in other studies, in which it was reported that a three-week exposure to 10 mg/kg BW/day of BPA caused liver damage with a consequent decrease in serum albumin [23]. Other authors such as Moon et al. [58] demonstrated this circumstance after exposure to doses of 50 µg/kg BW/day.

**Figure 6:**
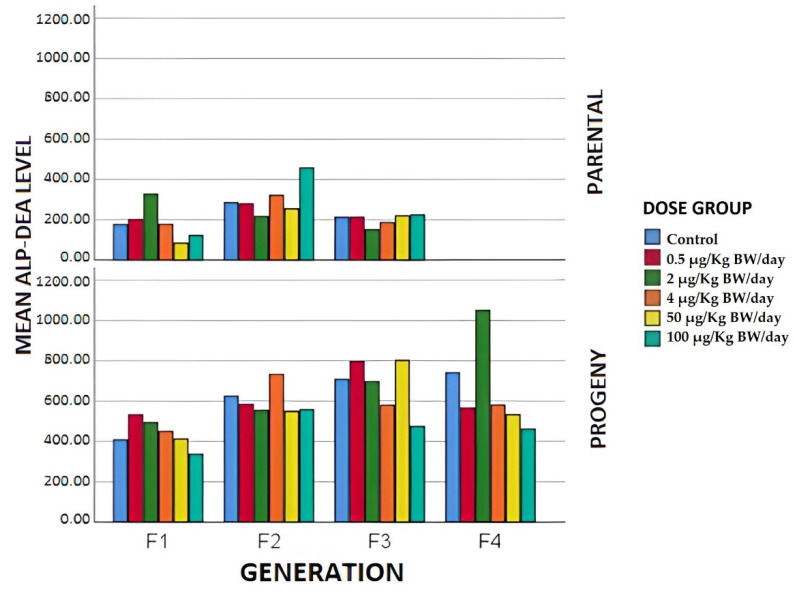
Mean alkaline phosphatase values expressed in U/L in the different generations of breeders and offspring in terms of the exposure group.

The liver damage suggested by the decrease in TOT PROT and ALB at the higher exposure doses could be better evidenced by the analysis of liver enzymes such as ALP-DEA, which in our results, showed significant differences (*p* < 0.05) with respect to the control, a typical behavior of a non-monotonic curve as reported in Figure 6, in which can be seen a change in the range of doses tried, very obvious both in F1 (with a clear inverted U) and in F3, where it can be seen as being U-shaped, and in the F2 breeder, where it is observed that the curve trajectory shows many inflection points.

The stronger activities of liver enzymes could be explained by the alteration in the permeability of the hepatocyte membrane induced by BPA; in which case, the cell membrane would lose its functional integrity, causing a cellular leakage of these enzymes into the bloodstream. This would be added to a decrease in the activity of endogenous enzymatic antioxidants, such as superoxide dismutase (SOD), glutathione peroxidase (GPx) and cytochrome P450 reductase (CYPR450) induced by BPA, which could increase the lipid peroxidation of the liver membrane, modifying its permeability [59].

In relation to the analysis of the lipid profile levels in serum, triglycerides, and total cholesterol (Table 4), the statistical analysis of our results did not reveal any significant differences (*p* > 0.05) between the exposure groups and the generations of parent animals, although the latter gave an increase in F2 and F3 at dose levels of 0.5 and 50 µg/kg BW/day, coinciding with the results of Lejonklon et al. [38]. This absence of significant differences concurred with the results obtained in the Clarity BPA study, where in neither of the two groups studied (stop-dose and continuous dose) were differences observed in triglyceride levels at the study doses (2.5, 25, 250, 2500, and 25,000 µg/kg BW/day). This would be an important factor to consider, since although BPA has a very fast metabolism, it could be thought that in continuous exposure to it alterations in triglyceride levels could occur (unlike in the stop group -dose). In fact, some animal studies have suggested that BPA could induce lipid abnormalities. In this sense, it has been demonstrated that early exposure to BPA increased circulating levels of TOT CHOL, TG, low-density lipoprotein cholesterol (LDL-C) and reduced levels of high-density lipoprotein cholesterol (HDL-C) [60].

In relation to urea and creatinine, our results (Table 4) for these two parameters did not reveal any significant differences between groups with respect to controls or between generations. Similarly, it was observed that the exposure time did not influence any of these modifications. Nevertheless, some animal studies have reported that exposure to BPA produces kidney damage, with an increase in serum creatinine and a decrease in urea levels. Although, its presence was determined at much higher exposure doses than those tested in this study [61].

### 3.2. Progeny

#### 3.2.1. Litter Weights

The mean values of the weights per litter were taken for the pups on days 0, 3, 7, 14, and 21 after birth (Table 5), showing significant differences (*p* < 0.05) in their weights between generations and exposure groups on day seven post-birth. As can be seen in Table 5, the mean weights in all the exposure groups were significantly lower in F3 compared to those in F0. Likewise, among the exposed groups, in F0 the highest mean values corresponded to groups exposed to higher dose levels, with significant differences (*p* < 0.05) between the latter and the control group and the groups with low doses (0.5, 2, and 4 µg/kg BW/day). In the first and third generation, the highest mean values corresponded to the litters from the group whose parents had been exposed to 2 µg/kg BW/day significantly higher values than those found in the control group. In the second generation, however, the group receiving 2 µg/kg BW/day was the one that gave significantly lower values than the rest of the exposed groups, including the control one. Our results differ from those obtained by other authors such as Cagen et al. [62] who, after exposing female mice during gestation to BPA concentrations of between 0.2 and 200 µg/kg BW/day, did not observe any effects on the pups’ growth in any of the dose ranges, thus concluding that ultra-low doses of chemical substances with a strong estrogenic capacity did not have any effects on offspring growth. Alternatively, other authors such as Bansal et al. [63], in a multigenerational study with exposure doses of 10 µg/kg BW/day, demonstrated an increase in weight in third generation litters.

##### Eye and Ear Opening Age

The mean values in days ranged from a maximum of 16 days in the group exposed to 100 µg/kg BW/day in F0, and a minimum of 13 days in the control groups, 0.5 and 2 µg/kg BW/day of F3. Our results did not reveal any differences (*p* > 0.05) between exposure groups and the study generation. This coincided with the results obtained by other authors, such as Ema et al. [64], who in a two-generation reproductive toxicity study in rats exposed orally to 0.2, 2, 20, or 200 µg/kg BW/day, did not obtain any significant differences in these parameters either.

#### 3.2.2. Biochemical Parameters

The mean values obtained for each of the parameters evaluated in the offspring are shown in Table 6.

The serum glucose levels (Figure 3) displayed significant differences between the exposure groups and the generations, with the highest glucose levels in the F1 whose mothers had been exposed to doses of 4 and 100 µg/kg BW/day. In F4, all the groups exposed to BPA showed elevated levels of glucose in serum with respect to the control, which may therefore suggest that there would be effects over successive generations (F4) of exposure to BPA on glucose metabolism at low doses, pointing to a possible predisposal to type 2 diabetes in the offspring of mothers exposed to BPA at the dose levels referred to [63].

In the case of perinatal exposure to BPA, some authors related this to an increase in fasting blood glucose, glucose intolerance, and insulin resistance in adult male offspring and in rats as a biomodel [65,66]. It has been seen that exposure to low doses of BPA during development causes hypersecretion of insulin in the offspring [67].

In this sense, authors such as Song et al. [50] exposed pregnant rats to BPA at concentrations of 1 and 10 µg/mL of BPA from day 6 of gestation to the end of lactation. Their results showed that perinatal exposure to 1 or 10 µg/mL BPA induced hyperglycemia with insulin resistance in the offspring in PND100, but only exposure to 10 µg/mL BPA already had similar effects in PND50. They concluded that, with their experimental range of BPA, the higher the dose to which the animals are exposed perinatally, the earlier its effect on glucose metabolism in the offspring. Dabeeret et al. [68] studied the effects of exposure to low doses of BPA (10 ppmx180 days) in the F0 generation of obese Wistar rats and its impact on the F1 generation, analyzing it on day 35 post-birth. It was observed that there were no differences in the serum glucose patterns of the F1 exposed in relation to the control, which would reveal the compensatory capacity of the organism, in which the glucose levels returned to the normal ranges of the species, although it would be interesting to elucidate if the tissue damage would be fully recovered.

However, Alonso-Magdalena et al. [53] demonstrated that low concentrations of BPA have long-term detrimental effects on glucose metabolism in mice during gestation and postpartum, as well as on their adult offspring. These authors demonstrated that low doses of BPA (10 and 100 µg/kg BW/day) administered subcutaneously to mothers during the days 9–16 of gestation caused a reduction in tolerance to glucose and an increase in insulin resistance, although these results were not observed until their offspring were six months of age. These results suggest that intrauterine exposure to BPA was associated with a decrease in glucose tolerance and an increase in insulin resistance in adult offspring. This is consistent with an effect of BPA on fetal programming that could predispose adult mice to type 2 diabetes and metabolic disorders.

In a two-generation study, Gengqi et al. [69] administered orally to pregnant female rats F0 a daily dose of 40 µg/kg BW/day during gestation and lactation, obtaining the F1 and F2 generations that were no longer exposed to BPA. At nine weeks after weaning, fasting blood glucose levels and serum insulin levels did not show any significant difference between controls and F2 offspring. These data differ from those obtained in our study, in which in F2, doses of 50 µg/kg BW/day gave significant increases in glucose levels with respect to the control group. This would be explained by the animals being continuously exposed to BPA, whereas those in the study by Gengqi et al. [69] after weaning had been left nine weeks without exposure to it. This compound has a fast metabolism, so that in that time it would have been metabolized and excreted from the body. In the same way, the effect that would occur after a continuous exposure to BPA, e.g., the increase in serum glucose would not happen when stopping exposure to EDC. It would be of interest to evaluate the pancreas of these animals to find out whether there is any type of persistent direct effect on it, and that is in some way made up for by the compensatory activity of the organism, so that this increase in serum glucose levels did not occur. Many unknown and important new studies remain to be made on these inadvertent actions, which translate into long-term effects on the health of exposed populations.

Regarding total protein levels (Figure 4), noteworthy results were obtained in F3, in which there was a significant decrease with respect to the control group in serum total protein levels in the group whose mothers were exposed to the highest dose of BPA. As can be seen in Figure 5, the serum albumin levels in the case of the offspring show a marked decrease in the case of the F2 and F3 individuals, with respect to the controls of the individuals whose parents were exposed to the highest doses of BPA. The F4 animals did not behave similarly, since it was the only group (2 µg/kg BW/day) presenting serum albumin levels below the control group. The reduction in TOT PROT in the highest levels of exposure doses could be due to liver damage. It could be useful to evaluate the liver in future studies, using histological and oxidative stress tests to investigate the effects of BPA on this tissue at different exposure dose levels in the different generations studied.

The TG, ALP-DEA, UREA, and CREAT levels did not exhibit any significant differences between exposure groups or generations in the case of offspring, and there were no bibliographic references to these parameters having been evaluated under similar study conditions to ours.

Some studies have determined different biochemical parameters after exposing laboratory animals to different BPA concentrations for a certain time. Based on the existing bibliography, this type of study would clearly indicate the effects of BPA on certain biochemical parameters such as glucose, triglycerides, cholesterol, creatinine, urea, alkaline phosphatase, and total proteins after the animals’ exposure to a BPA concentration for a certain time but, in fact, these populations are continuously exposed to this type of compound.

It would seem unrealistic to assume that it is currently possible to prevent exposure to BPA, so that there is a need for these types of studies which assess the effects on living organisms of BPA concentrations that are usually found both as an environmental and a food pollutant. On another level, multigenerational studies evaluating blood biochemistry after exposure to BPA are very rare [70,71]. Finally, a future line of research could be the exposure to cocktails of emerging pollutants [72], which could exert an additive or synergic effect, and could give rise to new risk reassessments.

## 4. Conclusions

The results obtained in this study showed that exposure to low BPA concentrations (0.5, 2, 4, 50, and 100 µg/kg BW/day) would apparently not have any effect on the reproduction parameters studied, or on those of the progeny growth. However, it was also determined that alterations in the biochemical parameters were produced over the generations exposed, and that the glucose, albumin, and total protein levels were modified. With respect to the glucose levels, it could be said that exposure to BPA causes a hyperglycemic effect, possibly due to an alteration in the metabolism of glucose in the pancreas. Further, the variations in the total protein and albumin levels would be elucidated by the BPA, inducing an alteration in the liver that would result in a modification mainly in the protein synthesis.

The absence at this moment of any safe alternatives to the employment of this chemical compound encourages the need for future studies that progress deeper into the knowledge of BPA toxicity. To our knowledge, this is the first report that investigates and identifies indirect biomarkers of effect over different generations of animals by analyzing blood biochemistry for this purpose. The recognition and use of these parameters as indirect biomarkers of effect could be of enormous use for biomonitoring the exposure to this endocrine disruptor as an environmental pollutant. New research lines could be established, contributing to the detection of possible early multigenerational effects of prolonged exposure to it at low concentrations that could occur in free-living animals, continuously and inadvertently exposed to emerging contaminants such as BPA.

## Figures and Tables

**Table 1:** Organization and target concentrations.

F0 Initial Mean Weigh ± SD	Dose Group
21.93 ± 3.26	CONTROL
23.20 ± 3.79	0.5 µg/Kg BW/day
22.75 ± 3.25	2 µg/kg BW/day
23.59 ± 3.66	4 µg/kg BW/day
21.58 ± 2.74	50 µg/kg BW/day
22.63 ± 3.48	100 µg/kg BW/day

**Table 2:** Percentage of pups that reach weaning alive by exposure group and parental generation.

Parental Generation	Dose Group	% Survival
F0	Control	100
	0.5 µg/kg BW/day	100
	2 µg/kg BW/day	90
	4 µg/kg BW/day	100
	50 µg/kg BW/day	90
	100 µg/kg BW/day	100
F1	Control	100
	0.5 µg/kg BW/day	93.3
	2 µg/kg BW/day	100
	4 µg/kg BW/day	100
	50 µg/kg BW/day	91.6
	100 µg/kg BW/day	88.8
F2	Control	100
	0.5 µg/kg BW/day	100
	2 µg/kg BW/day	100
	4 µg/kg BW/day	100
	50 µg/kg BW/day	100
	100 µg/kg/d	100
F3	Control	100
	0.5 µg/kg BW/day	90.9
	2 µg/kg BW/day	100
	4 µg/kg BW/day	100
	50 µg/kg BW/day	100
	100 µg/kg BW/day	100

**Table 3:** Litters sex ratio by generation and exposure group of their parents.

Generation	Dose Group	Mean% Males ± SD	Mean% Females ± SD	Sex Ratio
F0	Control	56.3 ± 0.088	43.7 ± 0.088	56:44
	0.5 µg/kg BW/day	47.9 ± 0.206	52.1 ± 0.206	48:52
	2 µg/kg BW/day	50.0 ± 0.181	50.0 ± 0.181	50:50
	4 µg/kg BW/day	70.8 ± 0.058	29.2 ± 0.589	71:29
	50 µg/kg BW/day	44.4 ± 0.181	55.6 ± 0.181	44:56
	100 µg/kg BW/day	75.0 ± 0.014	25.0 ± 0.111	75:25
F1	Control	55.0 ± 0.070	45.0 ± 0.070	55:45
	0.5 µg/kg BW/day	50.0 ± 0.101	50.0 ± 0.101	50:50
	2 µg/kg BW/day	53.6 ± 0.050	46.4 ± 0.050	54:46
	4 µg/kg BW/day	59.8 ± 0.037	40.2 ± 0.037	60:40
	50 µg/kg BW/day	28.3 ± 0.164	71.7 ± 0.164	28:72
	100 µg/kg BW/day	28.6 ± 0.404	71.4 ± 0.404	29:71
F2	Control	50.0 ± 0.303	50.0 ± 0.303	50:50
	0.5 µg/kg BW/day	45.0 ± 0.070	55.0 ± 0.070	45:55
	2 µg/kg BW/day	63.1 ± 0.286	36.9 ± 0.286	63:37
	4 µg/kg BW/day	57.1 ± 0.181	42.9 ± 0.181	57:43
	50 µg/kg BW/day	24.3 ± 0.060	75.7 ± 0.060	24:76
	100 µg/kg BW/day	66.0 ± 0.304	34.0 ± 0.304	66:34
F3	Control	46.4 ± 0.050	53.6 ± 0.050	46:54
	0.5 µg/kg BW/day	20.8 ± 0.058	79.2 ± 0.058	21:79
	2 µg/kg BW/day	35.7 ± 0.101	64.3 ± 0.101	36:64
	4 µg/kg BW/day	26.7 ± 0.094	73.3 ± 0.094	27:73
	50 µg/kg BW/day	87.5 ± 0.176	12.5 ± 0.176	87:13
	100 µg/kg BW/day	64.3 ± 0.303	35.7 ± 0.303	64:36

**Table 4:** Mean values ± standard deviations of the biochemical parameters in the different generations and exposure groups of breeding.

Parental Group and Generation		Glucose	Triglycerides	Total Protein	Cholesterol	Creatinine	Albumin	Urea	Alkaline Phosphatase
		Mean ± SD	Mean ± SD	Mean ± SD	Mean ± SD	Mean ± SD	Mean ± SD	Mean ± SD	Mean ± SD
F0	Control	261.175 ± 47.056	101.75 ± 11.814	57.200 ± 2.431	100.250 ± 14.407	0.375 ± 0.058	20.500 ± 1.290	46.450 ± 4.981	193.500 ± 44.003
	0.5 µg/kg BW/day	239.975 ± 15.462	92.25 ± 20.105	52.100 ± 1.283	84.500 ± 14.888	0.387 ± 0.027	19.000 ± 1.414	60.525 ± 18.717	321.000 ± 167.610
	2 µg/kg BW/day	235.800 ± 64.924	95.50 ± 36.336	56.200 ± 3.844	96.000 ± 16.206	0.393 ± 0.055	21.000 ± 2.160	46.950 ± 6.289	448.750 ± 309.303
	4 µg/kg BW/day	214.525 ± 20.475	110.500 ± 44.970	52.825 ± 1.203	111.500 ± 26.134	0.372 ± 0.036	19.250 ± 1.258	43.250 ± 5.952	378.250 ± 257.123
	50 µg/kg BW/day	180.567 ± 20.296	114.00 ± 40.632	52.733 ± 3.600	112.888 ± 11.372	0.470 ± 0.040	20.000 ± 2.000	65.067 ± 34.184	442.000 ± 359.654
	100 µg/kg BW/day	205.100 ± 35.522	80.750 ± 7.135	54.625 ± 1.858	95.250 ± 6.849	0.417 ± 0.032	19.000 ± 1.825	50.400 ± 14.886	427.000 ± 259.212
F1	Control	213.850 ± 16.205	103.000 ± 48.297	54.475 ± 6.367	84.000 ± 15.769	0.433 ± 0.095	21.000 ± 2.708	45.250 ± 10.883	176.500 ± 95.695
	0.5 µg/kg BW/day	234.200 ± 27.361	108.250 ± 39.601	57.625 ± 9.560	88.250 ± 17.613	0.412 ± 0.012	24.000 ± 5.354	47.225 ± 8.484	200.250 ± 151.856
	2 µg/kg BW/day	174.675 ± 42.685	52.500 ± 10.661	53.875 ± 2.590	81.250 ± 29.341	0.415 ± 0.017	20.500 ± 1.732	45.738 ± 14.399	328.250 ± 75.177
	4 µg/kg BW/day	231.550 ± 47.088	83.250 ± 40.729	53.175 ± 3.978	80.000 ± 29.040	0.375 ± 0.024	21.000 ± 2.708	42.225 ± 14.442	177.250 ± 102.024
	50 µg/kg BW/day	263.825 ± 67.902	89.500 ± 28.687	54.650 ± 6.739	90.000 ± 17.907	0.377 ± 0.046	22.750 ± 2.629	47.088 ± 10.334	183.250 ± 37.295
	100 µg/kg BW/day	244.725 ± 20.672	118.500 ± 38.613	52.775 ± 4.313	86.750 ± 22.706	0.405 ± 0.046	20.250 ± 0.957	50.735 ± 11.092	122.250 ± 28.052
F2	Control	236.200 ± 34.523	97.250 ± 20.287	54.725 ± 1.087	96.000 ± 8.286	0.450 ± 0.047	21.000 ± 0.816	46.088 ± 2.418	283.500 ± 51.137
	0.5 µg/kg BW/day	192.375 ± 10.665	84.250 ± 22.246	55.275 ± 3.472	100.250 ± 16.720	0.445 ± 0.026	21.000 ± 0.816	47.925 ± 4.742	277.750 ± 40.901
	2 µg/kg BW/day	268.925 ± 21.731	129.250 ± 23.796	54.725 ± 3.694	97.500 ± 26.664	0.385 ± 0.031	21.000 ± 1.414	38.450 ± 0.967	215.500 ± 24.569
	4 µg/kg BW/day	229.085 ± 24.832	51.250 ± 16.560	54.275 ± 3.551	78.750 ± 28.016	0.405 ± 0.024	21.750 ± 1.258	48.775 ± 7.511	319.750 ± 148.153
	50 µg/kg BW/day	266.800 ± 26.006	76.250 ± 17.346	52.750 ± 1.234	99.000 ± 13.976	5.555 ± 10.290	22.000 ± 1.414	41.700 ± 6.810	253.500 ± 33.669
	100 µg/kg BW/day	235.075 ± 33.947	65.000 ± 30.188	55.875 ± 3.099	98.500 ± 13.127	0.457 ± 0.046	16.500 ± 2.449	44.925 ± 5.573	456.250 ± 472.867
F3	Control	260.850 ± 37.989	138.750 ± 16.800	51.875 ± 3.703	96.750 ± 13.841	0.383 ± 0.032	20.000 ± 0.000	40.513 ± 5.559	211.750 ± 93.343
	0.5 µg/kg BW/day	326.100 ± 32.489	148.000 ± 77.816	52.375 ± 1.519	91.500 ± 11.000	0.360 ± 0.029	20.250 ± 0.500	39.438 ± 4.893	211.500 ± 60.467
	2 µg/kg BW/day	162.725 ± 42.410	105.000 ± 28.425	47.175 ± 14.590	83.500 ± 19.052	0.445 ± 0.143	20.000 ± 6.055	41.575 ± 15.491	149.750 ± 40.111
	4 µg/kg BW/day	286.400 ± 78.460	107.500 ± 26.501	53.200 ± 3.576	84.250 ± 26.600	0.377 ± 0.049	20.750 ± 1.500	43.650 ± 7.093	185.250 ± 31.063
	50 µg/kg BW/day	248.450 ± 72.523	116.000 ± 23.338	52.875 ± 1.504	95.500 ± 17.635	0.380 ± 0.029	20.500 ± 0.577	38.662 ± 7.214	219.250 ± 44.798
	100 µg/kg BW/day	324.675 ± 59.572	123.500 ± 23.187	51.275 ± 1.967	82.000 ± 19.866	0.358 ± 0.023	19.000 ± 0.816	28.413 ± 7.542	223.500 ± 34.317

**Table 5:** Mean weights ± standard deviations of the litters at days 0, 3, 7, 14, and 21 after the birth according to the parental generation of origin.

Parental Generation	Dose Group	PND0	PND3	PND7	PND14	PND21
		Mean ± SD	Mean ± SD	Mean ± SD	Mean ± SD	Mean ± SD
F0	Control	1.5 ± 0.169	1.956 ± 0.470	3.914 ± 0.168	6.642 ± 0.172	9.318 ± 0.414
	0.5 µg/kg BW/day	1.39 ± 0.628	2.55 ± 0.287	3.91 ± 0.497	6.43 ± 0.761	9.546 ± 0.205
	2 µg/kg BW/day	1.36 ± 0.127	1.47 ± 0.283	2.26 ± 0.332	5.805 ± 0.612	9.46 ± 0.547
	4 µg/kg BW/day	1.406 ± 0.760	1.63 ± 0.086	3.023 ± 0.709	5.598 ± 1.242	9.24 ± 0.596
	50 µg/kg BW/day	1.892 ± 0.127	2.72 ± 0.283	4.342 ± 0.332	5.899 ± 0.612	9.686 ± 0.547
	100 µg/kg BW/day	1.549 ± 0.684	2.694 ± 0.125	4.151 ± 0.291	6.904 ± 1.027	10.13 ± 0.552
F1	Control	1.295 ± 0.100	1.805 ± 0.035	3.407 ± 0.038	6.296 ± 0.309	9.49 ± 0.427
	0.5 µg/kg BW/day	1.285 ± 0.039	1.751 ± 0.376	3.299 ± 0.568	6.173 ± 0.482	8.97 ± 1.126
	2 µg/kg BW/day	1.317 ± 0.022	2.364 ± 0.283	4.278 ± 0.144	6.368 ± 0.313	8.91 ± 0.339
	4 µg/kg BW/day	1.174 ± 0.159	1.594 ± 0.037	3.357 ± 0.284	6.354 ± 0.279	8.94 ± 0.531
	50 µg/kg BW/day	1.308 ± 0.047	1.788 ± 0.130	3.64 ± 0.132	6.298 ± 0.271	8.86 ± 0.625
	100 µg/kg BW/day	1.457 ± 0.230	1.764 ± 0.290	3.496 ± 0.245	6.43 ± 0.438	9.34 ± 0.874
F2	Control	1.482 ± 0.232	1.964 ± 0.656	4.201 ± 0.037	6.373 ± 0.309	8.634 ± 0.210
	0.5 µg/kg BW/day	1.64 ± 0.613	2.422 ± 0.543	4.162 ± 0.257	5.798 ± 0.441	7.69 ± 0.503
	2 µg/kg BW/day	1.642 ± 0.247	1.903 ± 0.207	2.648 ± 0.438	5.862 ± 0.483	7.39 ± 0.606
	4 µg/kg BW/day	1.487 ± 0.127	1.851 ± 0.283	4.463 ± 0.332	6.433 ± 0.612	9.03 ± 0.547
	50 µg/kg BW/day	1.585 ± 0.024	2.39 ± 0.044	3.676 ± 0.653	7.479 ± 1.198	9.293 ± 0.402
	100 µg/kg BW/day	1.501 ± 0.033	2.282 ± 0.516	4.288 ± 0.017	6.488 ± 0.158	8.37 ± 1.041
F3	Control	1.441 ± 0.180	1.905 ± 0.565	2.75 ± 0.155	4.603 ± 1.159	6.37 ± 0.366
	0.5 µg/kg BW/day	1.625 ± 0.261	1.898 ± 0.188	2.974 ± 0.036	5.918 ± 0.494	7.28 ± 0.535
	2 µg/kg BW/day	1.329 ± 0.004	1.917 ± 0.072	3.719 ± 0.398	6.594 ± 0.152	7.42 ± 0.148
	4 µg/kg BW/day	1.208 ± 0.022	1.663 ± 0.227	2.502 ± 0.033	5.446 ± 0.030	6.98 ± 0.256
	50 µg/kg BW/day	1.273 ± 0.090	1.839 ± 0.067	2.639 ± 0.067	5.57 ± 0.127	6.74 ± 0.349
	100 µg/kg BW/day	1.26 ± 0.157	1.684 ± 0.025	2.583 ± 0.266	5.819 ± 0.178	7.15 ± 0.167

**Table 6:** Mean values ± standard deviations of the biochemical parameters in the different generations and exposure groups of offspring.

Offspring Group and Generation		Glucose	Tryglicerides	Total Protein	Cholesterol	Creatinine	Albumin	Urea	Alkaline Phosphatase
		Mean ± SD	Mean ± SD	Mean ± SD	Mean ± SD	Mean ± SD	Mean ± SD	Mean ± SD	Mean ± SD
F1	Control	265.283 ± 31.796	130.665 ± 51.852	44.734 ± 16.027	85.83 ± 33.700	0.335 ± 0.495	18.33 ± 3.295	39.2 ± 11.455	407.15 ± 38.395
	0.5 µg/kg BW/day	277.95 ± 38.537	108.25 ± 24.274	45.785 ± 1.718	87.33 ± 10.366	0.375 ± 0.007	18.83 ± 0.240	43.35 ± 0.777	531.15 ± 21.001
	2 µg/kg BW/day	243 ± 1.414	106 ± 11.313	42.5 ± 3.535	91.1 ± 1.555	0.325 ± 0.035	19 ± 1.414	40.6 ± 11.313	492 ± 9.899
	4 µg/kg BW/day	335.25 ± 42.431	138.5 ± 30.712	47.5 ± 5.280	77 ± 19.413	0.37 ± 1.870	20.5 ± 2.300	36.1 ± 10.381	448.5 ± 157.867
	50 µg/kg BW/day	203.967 ± 47.988	93.915 ± 39.011	40.04 ± 8.640	75.665 ± 33.000	0.315 ± 0.035	17 ± 2.828	46.3 ± 0.89	411.75 ± 18.031
	100 µg/kgBW/day	309.5 ± 42.431	118.5 ± 30.712	70.35 ± 5.280	110.75 ± 19.413	0.49 ± 1.870	34.5 ± 2.300	49.42 ± 10.381	335 ± 157.867
F2	Control	240.05 ± 16.334	92 ± 16.970	45.2 ± 6.788	95 ± 8.899	0.325 ± 0.007	20 ± 0.000	37.1 ± 1.979	624 ± 50.911
	0.5 µg/kg BW/day	235 ± 8.202	90.5 ± 4.949	52.15 ± 3.323	95.5 ± 6.363	0.355 ± 0.021	22 ± 1.414	41.4 ± 7.778	583.15 ± 15.344
	2 µg/kg BW/day	235.5 ± 7.778	97.5 ± 16.263	47.6 ± 1.979	84.3 ± 4.666	0.32 ± 0.028	20 ± 1.414	34.05 ± 3.747	552.5 ± 4.949
	4 µg/kg BW/day	204.8 ± 14.142	75 ± 1.414	55.5 ± 0.424	95.5 ± 6.363	0.39 ± 1.870	24 ± 0.000	52.3 ± 4.666	731.5 ± 54.447
	50 µg/kg BW/day	248.4 ± 20.364	74 ± 2.828	45 ± 7.071	81.5 ± 9.192	0.37 ± 0.084	18.5 ± 3.535	34.35 ± 6.717	548.5 ± 173.241
	100 µg/kg BW/day	214.25 ± 90.014	64 ± 36.769	53.55 ± 2.899	79.5 ± 24.748	0.37 ± 0.183	16.5 ± 7.778	33.55 ± 16.051	557 ± 200.818
F3	Control	325.95 ± 41.224	71 ± 25.455	51.8 ± 5.515	100 ± 33.941	0.37 ± 0.042	21.5 ± 2.121	35.55 ± 4.454	707.5 ± 127.986
	0.5 µg/kg BW/day	301.9 ± 21.920	55 ± 5.656	46.65 ± 1.767	90.5 ± 10.606	0.38 ± 0.042	19 ± 0.000	33.9 ± 2.262	795.5 ± 75.660
	2 µg/kg BW/day	268.5 ± 13.435	88.5 ± 41.719	47.95 ± 3.747	83.5 ± 4.949	0.34 ± 0.028	19 ± 1.414	39.9 ± 13.010	695.5 ± 164.755
	4 µg/kg BW/day	311.9 ± 42.431	94 ± 30.712	51.8 ± 5.280	98 ± 19.413	0.54 ± 1.870	21 ± 2.300	43 ± 10.381	579 ± 157.867
	50 µg/kg BW/day	236.3 ± 42.431	74 ± 30.712	48 ± 5.280	90 ± 19.413	0.23 ± 0.323	20 ± 2.300	32.8 ± 10.381	802 ± 157.867
	100 µg/kg BW/day	160.75 ± 128.622	31.5 ± 23.334	24.2 ± 18.384	49 ± 41.012	0.23 ± 1.870	15 ± 0.000	20.25 ± 4.313	473.5 ± 324.562
F4	Control	202.15 ± 4.454	131 ± 26.870	48.3 ± 2.262	78.5 ± 17.677	0.39 ± 0.056	19.5 ± 0.707	49.3 ± 0.565	740 ± 2.828
	0.5 µg/kg BW/day	271.05 ± 5.161	136.5 ± 10.606	52.05 ± 0.494	88 ± 16.970	0.37 ± 0.014	21.5 ± 0.707	46.7 ± 1.838	563.5 ± 21.920
	2 µg/kg BW/day	241.45 ± 20.011	82.5 ± 0.707	41.75 ± 1.343	73.5 ± 9.192	0.34 ± 0.042	18 ± 1.414	29.95 ± 2.757	1049.5 ± 27.577
	4 µg/kg BW/day	253.2 ± 4.949	113 ± 0.000	52.45 ± 1.626	72.5 ± 6.363	0.385 ± 0.007	22 ± 0.000	38 ± 3.959	579.5 ± 43.133
	50 µg/kg BW/day	285.9 ± 36.628	101.5 ± 14.849	49 ± 1.272	66.5 ± 14.849	0.4 ± 0.014	20 ± 0.000	37.8 ± 8.343	532 ± 9.899
	100 µg/kg BW/day	236.5 ± 51.123	125 ± 14.142	53.75 ± 3.889	79.5 ± 20.506	0.415 ± 0.007	21.5 ± 2.121	44.05 ± 10.960	461 ± 128.693

## Data Availability

Not applicable.

## Supplementary Materials

The following supporting information can be downloaded at: https://www.mdpi.com/article/10.3390/ani12030300/s1. Supplementary Table S1. Weight gains, feed, and water intake.
