# Supplementary material for: Analysis of Indirect Biomarkers of Effect after Exposure to Low Doses of Bisphenol A in a Study of Successive Generations of Mice
Source: Animals (Basel). 2022 Jan 26;12(3):300. doi: 10.3390/ani12030300 (PMC8833323; doi:10.3390/ani12030300)
Supplement: Supplementary file 1 [file animals-12-00300-s001.zip › animals-1506197-supplementary.pdf]

Supplementary Table S1: Weight gains, feed and water intake.

GENERATION	DOSE GROUP	SEX	DAILY WEIGH GAIN		DAILY FOOD INTAKE		DAILY WATER INTAKE	
			MEAN	SD	MEAN	SD	MEAN + SD	SD
F0	Control	MALE	,099	,056	5,331	,178	6,411	,171
		FEMALE	,111	,056	4,265	,178	4,054	,170
	0,5 µg/kg/d	MALE	,111	,056	5,792	,178	5,990	,170
		FEMALE	,102	,056	4,441	,178	4,429	,170
	2 µg/kg/d	MALE	,111	,056	4,324	,178	4,733	,170
		FEMALE	,130	,056	4,282	,178	3,694	,170
	4 µg/kg/d	MALE	,105	,056	5,082	,178	5,156	,170
		FEMALE	,122	,056	4,490	,178	3,984	,170
	50 µg/k/d	MALE	,082	,069	4,205	,217	5,076	,206
		FEMALE	,095	,056	4,301	,178	4,762	,170
	100 µg/kg/d	MALE	,114	,056	5,589	,178	5,557	,171
		FEMALE	,109	,056	4,633	,178	4,337	,170
F1	Control	MALE	,143	,107	4,735	,343	5,230	,327
		FEMALE	,137	,107	4,262	,343	4,513	,327
	0,5 µg/kg/d	MALE	,193	,107	4,977	,343	4,590	,327
		FEMALE	,100	,107	4,269	,343	3,868	,327
	2 µg/kg/d	MALE	,110	,107	5,617	,356	5,518	,340
		FEMALE	,128	,107	4,505	,343	4,022	,327
	4 µg/kg/d	MALE	,160	,107	4,890	,343	5,185	,327
		FEMALE	,101	,107	4,144	,343	4,300	,327
	50 µg/kg/d	MALE	,156	,107	4,610	,343	5,320	,333
		FEMALE	,115	,107	4,126	,343	3,924	,327
	100 µg/kg/d	MALE	,133	,107	4,487	,343	5,483	,327
		FEMALE	,121	,107	4,022	,343	4,213	,327
F2	Control	MALE	,110	,107	5,300	,343	4,952	,327
		FEMALE	,098	,107	4,387	,343	3,689	,327
	0,5 µg/kg/d	MALE	,122	,107	5,196	,343	5,043	,327
		FEMALE	,106	,107	4,379	,343	3,759	,327
	2 µg/kg/d	MALE	,127	,107	5,133	,343	4,672	,327
		FEMALE	,082	,107	4,463	,343	3,569	,327
	4 µg/kg/d	MALE	,108	,107	4,795	,343	4,874	,327
		FEMALE	,121	,107	4,752	,343	5,036	,327
	50 µg/kg/d	MALE	,056	,107	5,621	,343	5,134	,327
		FEMALE	,103	,107	4,461	,343	3,977	,327
	100 µg/kg/d	MALE	,085	,107	5,310	,343	5,221	,327
		FEMALE	,105	,107	3,092	,343	3,805	,327
F3	Control	MALE	,110	,107	6,137	,343	5,753	,327
		FEMALE	,089	,107	5,269	,343	3,969	,327
	0,5 µg/kg/d	MALE	,101	,107	6,943	,343	5,402	,327
		FEMALE	,064	,107	5,301	,343	3,834	,327
	2 µg/kg/d	MALE	,086	,107	5,703	,343	5,513	,327
		FEMALE	,112	,107	4,394	,343	4,307	,327
	4 µg/kg/d	MALE	,055	,107	7,312	,343	5,945	,327
		FEMALE	,091	,107	4,908	,343	3,883	,327
	50 µg/kg/d	MALE	,087	,107	7,607	,343	6,213	,327

	FEMALE	,131	,107	5,683	,343	3,641	,327
100 µg/kg/d	MALE	,068	,107	6,355	,343	5,301	,327
	FEMALE	,277	,107	4,884	,343	4,688	,327
